# Proposal of Allobrachymonas gen. nov. and three new combinations as replacement names for the illegitimate prokaryotic genus name Brachymonas, proposal of Allobosea gen. nov. and 12 new combinations as replacement names for the illegitimate prokaryotic genus name Bosea and proposal of Alloboseaceae fam. nov. as a replacement name for the illegitimate family name Boseaceae

**DOI:** 10.1099/ijsem.0.007072

**Published:** 2026-02-05

**Authors:** Umakant Bhoopati Deshmukh, Aharon Oren

**Affiliations:** 1 Department of Botany, Institution of Higher Learning, Research and Specialized Studies Centre (affiliated with Gondwana University, Gadchiroli), Janata Mahavidyalaya, Chandrapur 442 401, India; 2 Department of Plant and Environmental Sciences, The Institute of Life Sciences, The Hebrew University of Jerusalem, Edmond J. Safra Campus, Jerusalem 9190401, Israel

**Keywords:** *Bosea*, *Boseaceae*, *Brachymonas*, illegitimate names

## Abstract

The prokaryotic generic names *Brachymonas* Hiraishi *et al*. 1995 and *Bosea* Das *et al*. 1996 are illegitimate because they are later homonyms of the genus names *Brachymonas* Grassé 1952 (Protozoa, Polymastigidae) and *Bosea* He and Qian 1979 (a fossil dinoflagellate) [Principle 2 and Rule 51b(4) of the International Code of Nomenclature of Prokaryotes (ICNP)]. We therefore propose the replacement names *Allobrachymonas* gen. nov., *Allobrachymonas chironomi* comb. nov., *Allobrachymonas denitrificans* comb. nov., *Allobrachymonas wangyanguii* comb. nov., *Allobosea* gen. nov., *Allobosea beijingensis* comb. nov., *Allobosea caraganae* comb. nov., *Allobosea eneae* comb. nov., *Allobosea lathyri* comb. nov., *Allobosea lupini* comb. nov., *Allobosea massiliensis* comb. nov., *Allobosea minatitlanensis* comb. nov., *Allobosea psychrotolerans* comb. nov., *Allobosea robiniae* comb. nov., *Allobosea rubneri* comb. nov., *Allobosea spartocytisi* comb. nov., *Allobosea thiooxidans* comb. nov., *Allobosea vaviloviae* comb. nov., *Allobosea vestrisii* comb. nov. and *Alloboseaceae* fam. nov.

## Introduction

The prokaryotic genus *Brachymonas* (*Comamonadaceae*, *Burkholderiales, Betaproteobacteria*, *Pseudomonadota*) was described by Hiraishi *et al*. in 1995 [1]. The genus currently contains three species with validly published names. The generic name *Brachymonas* is a homonym of *Brachymonas* Grassé 1952 [2], a subgenus of *Monocercomonoides* (Polymastigidae, Metamonada, Protozoa), elevated to the rank of genus in 2018 [3]. Based on Principle 2 and Rule 51b(4) of the International Code of Nomenclature of Prokaryotes (ICNP) [4] and the version of the International Code of Nomenclature of Bacteria in effect at the time of publication [5], the name *Brachymonas* proposed for a prokaryotic genus is illegitimate.

The ICNP offers two possibilities to resolve the illegitimacy of a name: by replacing an illegitimate name with a new legitimate one, or by addressing a request for an opinion to the Judicial Commission to obtain an exception allowing the illegitimate name to be conserved (see [6,7] for recent examples). As the replacements proposed in this manuscript are not expected to cause errors or confusion, we prefer the first option here. In accordance with Rule 54, we here propose replacement names, conserving the meaning of the illegitimate names as originally published.

## Description of *Allobrachymonas* Deshmukh and Oren gen. nov.

*Allobrachymonas* (Al.lo.bra.chy.mo’nas. Gr. masc. adj. *allos*, other; N.L. fem. n. *Brachymonas* (‘a small short unit’), a bacterial genus; N.L. fem. n. *Allobrachymonas*, another *Brachymonas*).

Earlier illegitimate homotypic synonym: *Brachymonas* Hiraichi *et al*. 1995 [1].

The description of the genus is as given for *Brachymonas* [1,8].

The type species is *Allobrachymonas denitrificans* (Hiraishi *et al*. 1995) Deshmukh and Oren.

## Description of *Allobrachymonas chironomi* (Halpern *et al*. 2009) Deshmukh and Oren comb. nov.

*Allobrachymonas chironomi* (chi.ro’no.mi. N.L. gen. n. *chironomi*, of *Chironomus*, named after the non-biting midge insect of the genus *Chironomus* (Chironomidae, Diptera) from which the type strain was isolated).

Basonym: *Brachymonas chironomi* Halpern *et al*. 2009 [8].

The description of the species is as given for *Brachymonas chironomi* [8].

The type strain is AImA4 (=DSM 19884=LMG 24400), isolated from a chironomid egg mass sampled from a waste stabilization pond in northern Israel.

The 16S rRNA gene sequence accession number of the type strain is EU346912.

The genome sequence of the type strain [9] can be accessed from GenBank as GCA_000374625.

## Description of *Allobrachymonas denitrificans* (Hiraishi *et al.* 1995) Deshmukh and Oren comb. nov.

*Allobrachymonas denitrificans* (de.ni.tri’fi.cans. N.L. fem. part. adj. *denitrificans*, denitrifying).

Basonym: *Brachymonas denitrificans* Hiraishi *et al*. 1995 [1,10].

The description of the species is as given for *Brachymonas denitrificans* [1].

The type strain is AS-P1 (=CCUG 45365=DSM 15123=JCM 9216=LMG 31428), isolated from soybean curd waste sludge in Japan.

The 16S rRNA gene sequence accession number of the type strain is D14320.

The genome sequence of the type strain can be accessed from GenBank as GCA_900110225.

## Description of *Allobrachymonas wangyanguii* (Gao *et al.* 2025) Deshmukh and Oren comb. nov.

*Allobrachymonas wangyanguii* (wang.yan.gui’i. N.L. gen. n. *wangyanguii*, named in honour of Professor Yangui Wang, microbiologist at the Department of Microbiology, Shanxi Medical University, in recognition of his outstanding contributions to medical microbiology education).

Basonym: *Brachymonas wangyanguii* Gao *et al*. 2025 [11].

The description of the species is as given for *Brachymonas wangyanguii* [11].

The type strain is G13 (=GDMCC 1.4691=JCM 37746), isolated from an *in situ* coal block sample obtained from Xinzhou, Shanxi, China.

The 16S rRNA gene sequence accession number of the type strain is PP212021. Strain G34 (16S rRNA gene sequence accession no. PQ632505) is a second strain of the species.

The genome sequence of the type strain can be accessed from GenBank as GCA_045055315.

The name of a fourth species, ‘*Brachymonas petroleovorans*’ (16S rRNA gene sequence accession no. AY275432) [12], was not validly published, and no cultures appear to be available for study.

The prokaryotic genus *Bosea* (*Boseaceae*, *Hyphomicrobiales, Alphaproteobacteria*, *Pseudomonadota*) was described by Das *et al*. in 1996 [13]. The genus currently contains 14 species with validly published names. The generic name *Bosea* is a homonym of *Bosea* He and Qian 1979, a fossil dinoflagellate [14]. Based on Principle 2 and Rule 51b(4) of the ICNP [4] and the version of the International Code of Nomenclature of Bacteria in effect at the time of publication [5], the name *Bosea* proposed for a prokaryotic genus is illegitimate. It is also a homonym of a genus of higher plants (*Bosea* Linnaeus 1753, family *Amaranthaceae*). We here propose replacement names, conserving the meaning of the illegitimate names as originally published.

## Description of *Allobosea* Deshmukh and Oren gen. nov.

*Allobosea* (Al.lo.bo’se.a. Gr. masc. adj. *allos*, other; N.L. fem. n. *Bosea* (‘named in honour of J.C. Bose, the founder of the Bose Institute, where the type species *Bosea thiooxidans* was isolated’), a bacterial genus; N.L. fem. n. *Allobosea*, another *Bosea*).

Earlier illegitimate homotypic synonym: *Bosea* Das *et al*. 1996 [13].

The description of the genus is as given for *Bosea* [13,15].

The type species is *Allobosea thiooxidans* (Das *et al*. 1996) Deshmukh and Oren.

## Description of *Allobosea beijingensis* (Gu *et al.* 2024) Deshmukh and Oren comb. nov.

*Allobosea beijingensis* (bei.jing.en’sis. N.L. fem. adj. *beijingensis*, pertaining to Beijing, where the type strain was cultured and isolated).

Basonym: *Bosea beijingensis* Gu *et al*. 2024 [16].

The description of the species is as given for *Bosea beijingensis* [16].

The type strain is REN20 (=GDMCC 1.2894=JCM 35118), isolated from a baijiu mash sample from Sichuan, PR China.

The 16S rRNA gene sequence accession number of the type strain is OR405985.

The genome sequence of the type strain can be accessed from GenBank as GCA_030758975.

## Description of *Allobosea caraganae* (Sazanova *et al.* 2019) Deshmukh and Oren comb. nov.

*Allobosea caraganae* (ca.ra.ga’nae. N.L. gen. n. *caraganae*, of the leguminous plant genus *Caragana*, from which this species was first isolated).

Basonym: *Bosea caraganae* Sazanova *et al*. 2019 [17].

The description of the species is as given for *Bosea caraganae* [17].

The type strain is LMG 31125 (=RCAM 4680), isolated from root nodules of the relict legume *Caragana jubata* from the shore of Lake Khuvsgul, Mongolia. Strain RCAM 04685 is a second strain of the species.

The 16S rRNA gene sequence accession number of the type strain is MH633716.

The genome sequence of the type strain can be accessed from GenBank as GCA_003351345.

## Description of *Allobosea eneae* (La Scola *et al.* 2003) Deshmukh and Oren comb. nov.

*Allobosea eneae* (e’ne.ae. N.L. gen. n. *eneae*, of Enea, to honour Maryse Enea, a technician in the Unité des Rickettsies, for her many contributions to the isolation of obligate intracellular bacteria, especially *Rickettsiaceae*).

Basonym: *Bosea eneae* La Scola *et al*. 2003 [15].

The description of the species is as given for *Bosea eneae* [15].

The type strain is 34614 (=CCUG 43111=CIP 106338=DSM 21596), isolated from the water supply of the La Timone Hospital Centre, Marseille, France.

The 16S rRNA gene sequence accession number of the type strain is AF288300.

The genome sequence of the type strain is not available.

## Description of *Allobosea lathyri* (De Meyer and Willems 2012) Deshmukh and Oren comb. nov.

*Allobosea lathyri* (la.thy’ri. N.L. gen. n. *lathyri*, of the leguminous plant genus *Lathyrus,* from which this species was first isolated).

Basonym: *Bosea lathyri* De Meyer and Willems 2012 [18].

The description of the species is as given for *Bosea lathyri* [18].

The type strain is R-46060 (=CCUG 61247=DSM 26656=LMG 26379), isolated from root nodules of *Lathyrus latifolius* in Flanders, Belgium.

The 16S rRNA gene sequence accession number of the type strain is FR774993.

The genome sequence of the type strain can be accessed from GenBank as GCA_900108245.

## Description of *Allobosea lupini* (De Meyer and Willems 2012) Deshmukh and Oren comb. nov.

*Allobosea lupini* (lu.pi’ni. L. gen. n. *lupini*, of the leguminous plant genus *Lupinus,* from which this species was first isolated).

Basonym: *Bosea lupini* De Meyer and Willems 2012 [18].

The description of the species is as given for *Bosea lupini* [18].

The type strain is R-45681 (=CCUG 61248=DSM 26673=LMG 26383), isolated from root nodules of *Lupinus polyphyllus* in Flanders, Belgium.

The 16S rRNA gene sequence accession number of the type strain is FR774992.

The genome sequence of the type strain can be accessed from GenBank as GCA_900109525.

## Description of *Allobosea massiliensis* (La Scola *et al.* 2003) Deshmukh and Oren comb. nov.

*Allobosea massiliensis* (mas.si.li.en’sis. L. fem. adj. *massiliensis*, pertaining to Massilia, the ancient Roman name of Marseille, France, where the type strain was isolated).

Basonym: *Bosea massiliensis* La Scola *et al*. 2003 [15].

The description of the species is as given for *Bosea massiliensis* [15].

The type strain is 63287 (=CCUG 43117=CIP 106336=DSM 21079), isolated from the water supply of the La Timone Hospital Centre, Marseille, France.

The 16S rRNA gene sequence accession number of the type strain is AF288309.

The genome sequence of the type strain is not available.

## Description of *Allobosea minatitlanensis* (Ouattara *et al.* 2003) Deshmukh and Oren comb. nov.

*Allobosea minatitlanensis* (mi.na.ti.tla.nen’sis. N.L. fem. adj. *minatitlanensis*, of Minatitlán, a town in the south of Veracruz state in Mexico, where the anaerobic sludge of a pilot-scale reactor was sampled to inoculate the lab-scale upflow anaerobic sludge blanket reactor from which the type strain was isolated).

Basonym: *Bosea minatitlanensis* Ouattara *et al*. 2003 [19].

The description of the species is as given for *Bosea minatitlanensis* [19].

The type strain is AMX 51 (=ATCC 700918=CIP 106457=DSM 13099), isolated as a transient micro-organism from anaerobic sludge of a lab-scale upflow anaerobic sludge blanket reactor treating the petrochemical wastewater of a purified terephthalic acid plant in Minatitlán, Mexico.

The 16S rRNA gene sequence accession number of the type strain is AF273081.

The genome sequence of the type strain can be accessed from GenBank as GCF_025209975.

## Description of *Allobosea psychrotolerans* (Albert *et al.* 2019) Deshmukh and Oren comb. nov.

*Allobosea psychrotolerans* (psy.chro.to’le.rans. Gr. masc. adj. *psychros*, cold; L. pres. part. *tolerans*, tolerating; N.L. fem. part. adj. *psychrotolerans*, cold-tolerating).

Basonym: *Bosea psychrotolerans* Albert *et al*. 2019 [20].

The description of the species is as given for *Bosea psychrotolerans* [20].

The type strain is 1131 (=LMG 30034=NRRL B-65405), isolated from a Lake Michigan potable water sample in south-eastern Wisconsin.

The 16S rRNA gene sequence accession number of the type strain is KX355625.

The genome sequence of the type strain can be accessed from GenBank as GCF_002917105.

## Description of *Allobosea robiniae* (De Meyer and Willems 2012) Deshmukh and Oren comb. nov.

*Allobosea robiniae* (ro.bi’ni.ae. N.L. gen. n. *robiniae*, of *Robinia*, the leguminous plant genus from which this species was first isolated).

Basonym: *Bosea robiniae* De Meyer and Willems 2012 [18].

The description of the species is as given for *Bosea robinae* [18].

The type strain is R-46070 (=CCUG 61249=DSM 26672=LMG 26381), isolated from root nodules of *Robinia pseudoacacia* in Flanders, Belgium.

The 16S rRNA gene sequence accession number of the type strain is FR774994.

The genome sequence of the type strain can be accessed from GenBank as GCA_900102525.

## Description of *Allobosea rubneri* (Stoll *et al.* 2024) Deshmukh and Oren comb. nov.

*Allobosea rubneri* (rub’ne.ri. N.L. gen. n. *rubneri*, of Rubner, referring to Max Rubner, a German physiologist, after whom the Max Rubner-Institute was named, and where the type strain was isolated).

Basonym: *Bosea rubneri* Stoll *et al*. 2024 [21,22].

The description of the species is as given for *Bosea rubneri* [21].

The type strain is ZW___T025 (=DSM 116094=LMG 33093), isolated from a bulb of an onion hybrid race (*Allium cepa* var. Hytech F1) grown in Kleinhohenheim, Germany.

The 16S rRNA gene sequence accession number of the type strain is OR512845.

The genome sequence of the type strain can be accessed from GenBank as GCF_032464875.

## Description of *Allobosea spartocytisi* (Pulido-Suárez *et al.* 2023) Deshmukh and Oren comb. nov.

*Allobosea spartocytisi* (spar.to.cy’ti.si. N.L. gen. n. *spartocytisi*, of the generic name *Spartocytisus*, referring to the source of this bacterium’s isolation, root nodules of *Spartocytisus supranubius*).

Basonym: *Bosea spartocytisi* Pulido-Suárez *et al*. 2023 [23,24].

The description of the species is as given for *Bosea spartocytisi* [23].

The type strain is SSUT16 (=CECT 30526=HAMBI 3759=LMG 32510), isolated from a root nodule of *S. supranubius*, Teide National Park, Tenerife, Canary Islands.

The 16S rRNA gene sequence accession number of the type strain is MT186807.

The genome sequence of the type strain can be accessed from GenBank as GCF_014764685.

## Description of *Allobosea thiooxidans* (Das *et al.* 1996) Deshmukh and Oren comb. nov.

*Allobosea thiooxidans* (thi.o.o’xi.dans. Gr. neut. n. *theion*, sulfur; N.L. v. *oxido*, to oxidize; from Gr. masc. adj. *oxys*, sour, acid; N.L. fem. part. adj. *thiooxidans*, oxidizing sulfur).

Basonym: *Bosea thiooxidans* Das *et al*. 1996 [13].

The description of the species is as given for *Bosea thiooxidans* [13].

The type strain is BI 42 (=ATCC 700366=DSM 9653), isolated from agricultural soil around Calcutta, India.

The 16S rRNA gene sequence accession number of the type strain is AJ250796.

The genome sequence of the type strain can be accessed from GenBank as GCA_900168195.

## Description of *Allobosea vaviloviae* (Safronova *et al.* 2015) Deshmukh and Oren comb. nov.

*Allobosea vaviloviae* (va.vi.lo’vi.ae. N.L. gen. n. *vaviloviae*, of the leguminous plant genus *Vavilovia*, from which the type strain was isolated).

Basonym: *Bosea vaviloviae* Safronova *et al*. 2015 [25,26].

The description of the species is as given for *Bosea vaviloviae* [25].

The type strain is Vaf-18 (=LMG 28367=RCAM 2129), isolated from the nodules of *Vavilovia formosa* plants growing in the hard-to-reach mountainous region of the North Ossetian State Natural Reserve (north Caucasus, Russian Federation).

The 16S rRNA gene sequence accession number of the type strain is KJ721000.

The genome sequence of the type strain can be accessed from GenBank as GCA_001741865.

## Description of *Allobosea vestrisii* (La Scola *et al.* 2003) Deshmukh and Oren comb. nov.

*Allobosea vestrisii* (ves.tri’si.i. N.L. gen. n. *vestrisii*, of Vestris, to honour Guy Vestris, a technician in the Unité des Rickettsies, for his many contributions to the isolation of obligate intracellular bacteria, especially *Rickettsiaceae*).

Basonym: *Bosea vestrisii* La Scola *et al*. 2003 [15].

The description of the species is as given for *Bosea vestrisii* [15].

The type strain is 34635 (=CCUG 43114=CIP 106340=DSM 21229), isolated from the water supply of the La Timone Hospital Centre, Marseille, France.

The 16S rRNA gene sequence accession number of the type strain is AF288306.

The genome sequence of the type strain is not available.

## Description of *Alloboseaceae* fam. nov.

*Alloboseaceae* (Al.lo.bo.se.a.ce’ae. N.L. fem. n. *Allobosea*, a bacterial genus; -*aceae*, ending to denote a family; N.L. fem. pl. n. *Alloboseaceae*, the *Allobosea* family).

Earlier illegitimate homotypic synonym: *Boseaceae* Hördt *et al*. 2000 [27,28].

The description of the family is as given for the family *Boseaceae* [27].

The type genus is *Allobosea* Deshmukh and Oren.

## Abbreviations

ICNP: International Code of Nomenclature of Prokaryotes
